# Disentangling the impacts of heat wave magnitude, duration and timing on the structure and diversity of sessile marine assemblages

**DOI:** 10.7717/peerj.863

**Published:** 2015-03-26

**Authors:** Dan A. Smale, Anna L.E. Yunnie, Thomas Vance, Stephen Widdicombe

**Affiliations:** 1 Marine Biological Association of the United Kingdom, The Laboratory, Citadel Hill, Plymouth, UK; 2 PML Applications Ltd, Prospect Place, Plymouth, UK; 3 Plymouth Marine Laboratory, Prospect Place, Plymouth, UK

**Keywords:** Marine biodiversity, Warming, Marine invertebrates, Temperature variability, Climate change

## Abstract

Extreme climatic events, including heat waves (HWs) and severe storms, influence the structure of marine and terrestrial ecosystems. Despite growing consensus that anthropogenic climate change will increase the frequency, duration and magnitude of extreme events, current understanding of their impact on communities and ecosystems is limited. Here, we used sessile invertebrates on settlement panels as model assemblages to examine the influence of HW magnitude, duration and timing on marine biodiversity patterns. Settlement panels were deployed in a marina in southwest UK for ≥5 weeks, to allow sufficient time for colonisation and development of sessile fauna, before being subjected to simulated HWs in a mesocosm facility. Replicate panel assemblages were held at ambient sea temperature (∼17 °C), or +3 °C or +5 °C for a period of 1 or 2 weeks, before being returned to the marina for a recovery phase of 2–3 weeks. The 10-week experiment was repeated 3 times, staggered throughout summer, to examine the influence of HW timing on community impacts. Contrary to our expectations, the warming events had no clear, consistent impacts on the abundance of species or the structure of sessile assemblages. With the exception of 1 high-magnitude long-duration HW event, warming did not alter not assemblage structure, favour non-native species, nor lead to changes in richness, abundance or biomass of sessile faunal assemblages. The observed lack of effect may have been caused by a combination of (1) the use of relatively low magnitude, realistic heat wave treatments compared to previous studies (2), the greater resilience of mature adult sessile fauna compared to recruits and juveniles, and (3) the high thermal tolerance of the model organisms (i.e., temperate fouling species, principally bryozoans and ascidians). Our study demonstrates the importance of using realistic treatments when manipulating climate change variables, and also suggests that biogeographical context may influence community-level responses to short-term warming events, which are predicted to increase in severity in the future.

## Introduction

Ecosystems the world over have responded to climate change, with major implications for humanity’s use of ecological goods and services (Rosenzweig et al., 2008; IPCC, 2014). Links between a changing climate and shifts in species distributions and the structure of communities and ecosystems have been documented convincingly for many taxa across many regions (Parmesan & Yohe, 2003; Wernberg et al., 2011; Pinsky et al., 2013; Poloczanska et al., 2013). In conjunction with gradual warming trends, discrete extreme climatic events are increasing in frequency and intensity as a consequence of anthropogenic climate change (IPCC, 2012; Coumou, Robinson & Rahmstorf, 2013). As such, understanding and predicting biological responses to ‘events’, rather than ‘trends’, is becoming increasingly important, although event-based research still lags behind trend-based work (Jentsch, Kreyling & Beierkuhnlein, 2007; Thompson et al., 2013). It is clear that discrete climatic events can drive step-wise changes in species’ distributions and, ultimately, ecosystem structure and functioning. Storms, floods and heat waves, for example, can have catastrophic effects on both marine and terrestrial ecosystems (Jentsch, Kreyling & Beierkuhnlein, 2007; IPCC, 2012), with substantial socio-economic ramifications.

Despite growing appreciation of the importance of extreme events in determining ecosystem structure (Jentsch, Kreyling & Beierkuhnlein, 2007; Thompson et al., 2013), the vast majority of knowledge stems from terrestrial research, even though marine ecosystems provide myriad of ecological goods and services, including nutrient cycling, provision of food and other resources, biogenic coastal defence and climate regulation. Marine ecosystems, like their terrestrial counterparts, are affected by extreme climatic events, including heat waves (Garrabou et al., 2009; Wernberg et al., 2013), cold snaps (Firth, Knights & Bell, 2011), storms (Dayton & Tegner, 1984; De’ath et al., 2012) and floods (Fabricius et al., 2014), which are driven by complex oceanographic processes such as ENSO, as well as interactions across the air-sea and land-sea interfaces. In shallow marine habitats, the number of days of anomalously high seawater temperatures has increased along 30% of the world’s coastlines in the last 30 years (Lima & Wethey, 2012). The recent European heat waves (‘HWs’) of 2003 and 2006, for example, induced widespread mortality, shifts in species’ distributions and declines in local biodiversity in the Mediterranean Sea (Garrabou et al., 2009; Lejeusne et al., 2009; Marba & Duarte, 2010). Similarly, a record-breaking marine HW in the southeast Indian Ocean in 2011 caused major alterations to the structure of benthic ecosystems and loss of habitat-forming species along the west Australian coastline (Moore et al., 2012; Smale & Wernberg, 2013; Wernberg et al., 2013). Prolonged periods of extremely high seawater temperatures affect processes across all biological scales, from genes (Bergmann et al., 2010) to whole organisms (Diaz-Almela, Marba & Duarte, 2007) to ecosystems (Wernberg et al., 2013).

There is a pressing need to understand how marine HWs affect the ecological performance of benthic marine organisms and how climate variability influences species interactions and community dynamics (Thompson et al., 2013). Few studies have simulated short-term climate variability in marine environments (but see Smale et al., 2011; Kim & Micheli, 2013; Kordas et al., in press) and even fewer have disentangled mean effects of climate variables from increased variability (but see Benedetti-Cecchi et al., 2006). More generally, experiments in marine climate change ecology have tended to focus on a few species in isolation, most often within a laboratory setting, rather than on suites of multiple species interacting with each other and the natural environment (Wernberg, Smale & Thomsen, 2012). Understanding how temperature variability, in conjunction with mean increases in temperature, will affect species interactions and community dynamics will improve our ability to predict, and plan for, ecological responses to climate change (Kordas, Harley & O’Connor, 2011; Wernberg, Smale & Thomsen, 2012).

Sessile marine assemblages (i.e., marine organisms attached to hard substrata) have proved useful experimental models for investigating the relative importance of both physical (Piola & Johnston, 2008) and biological processes (Smale, 2013). Within the context of marine climate change, several studies have manipulated temperature on or around settlement panels colonised by sessile marine invertebrates, to examine their response to warming (Sorte, Fuller & Bracken, 2010; Smale et al., 2011; Kim & Micheli, 2013; Kordas et al., in press). Here we developed this approach further by conducting the most complex warming experiment to date, whereby 3 key properties of marine HWs (magnitude, duration and timing) were manipulated and mature sessile invertebrate assemblages were subjected to realistic HW simulations. Based on existing knowledge we proposed and tested the following hypotheses: (1) high magnitude, long duration HWs will be detrimental to some marine taxa and cause shifts in community structure (Garrabou et al., 2009; Wernberg et al., 2013); (2) high magnitude, long duration HWs will be less detrimental (and perhaps beneficial) to non-native species compared with native species, as non-native species often exhibit wide thermal tolerances and high resilience to environmental stress (Stachowicz et al., 2002; Sorte, Fuller & Bracken, 2010; Sorte, Williams & Zerebecki, 2010; Diez et al., 2012), and; (3) these patterns will be broadly consistent across different HWs that occur during seasonal thermal maxima (i.e., during summer, HW ‘timing’ will be less important than HW magnitude and duration).

## Material and Methods

### Study site and sessile assemblages

Experimental assemblages were grown under natural conditions on panels deployed at Torquay Marina, southwest U.K. (50°27′31.44″N, 3°31′45.20″W). The study site is relatively unimpacted by freshening events and supports a high abundance and diversity of sessile invertebrate fauna. Submerged hard surfaces are colonised by a rich marine fauna, dominated by ascidians (e.g., *Ciona intestinalis*, *Diplosoma listerianum*, *Botryllus schlosseri*), bryozoans (e.g., *Electra pilosa*, *Cryptosula pallasina*) and polychaetes (e.g., *Spirobranchus* sp.). Filamentous algae and macroalgae are also common on vertical and upward-facing surfaces that receive sufficient light. Several non-native species have also been recorded at the study site (e.g., the bryozoans *Watersipora subtorquata* and *Bugula neritina*, the ascidians *Asterocarpa humilus* and *Corella eumyota*).

### Experimental design

**Figure 1:**
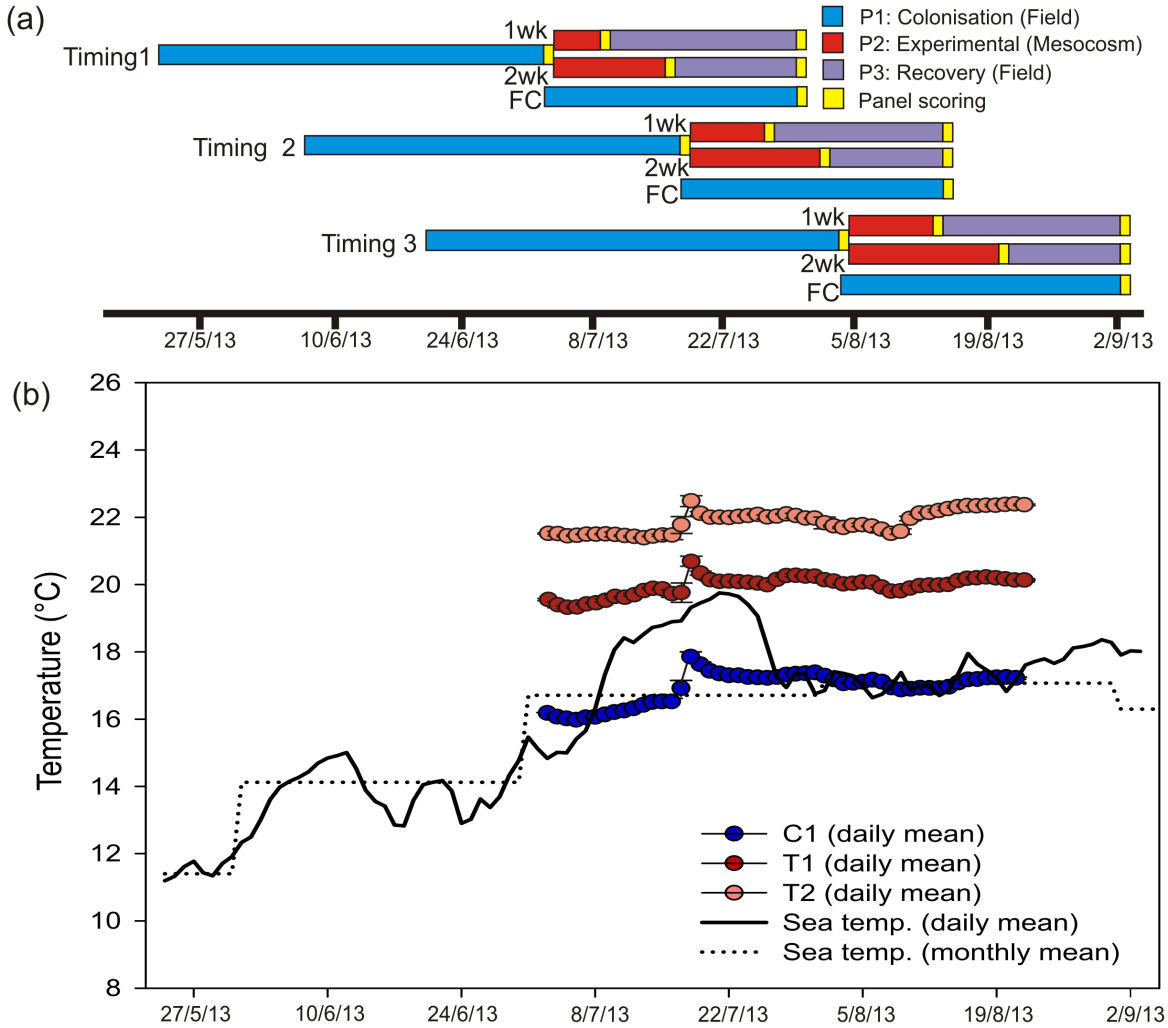
Heat wave simulations and experimental design. Experimental design and temperature treatments. (A) Coloured bars show timings and durations of experimental phases within each HW Timing. For each Timing, a set of Field Controls (FC) remained *in situ*. (B) Time series indicating sea temperature at the study site (daily means are based on 72 records per day, taken every 20 min; monthly mean generated from daily temperatures recorded over 3 preceding years), as well as mean daily temperatures (*n* = 5 mesocosm tanks ± SD) for each HW Magnitude treatment during the experimental period.

The experiment comprised of 3 phases; (1) a ‘colonisation’ phase in the field, (2) an ‘experimental’ phase within mesocosms of either 1 or 2 weeks in duration and (3) a final ‘recovery’ phase in the field (Fig. 1). The experiment was initiated at 3 different times throughout the summer of 2013 to investigate the effects of HW timing on ecological responses. For Phase 1, 36 settlement panels (20 × 20 cm, constructed from black double-skinned sheets of polypropylene, ‘Correx’) were attached to a fibreglass reinforced plastic (FRP) grid in 6 rows of 6 panels (Fig. S1). The FRP grid (*L* = 148 cm, *W* = 121 cm, *D* = 2.5 cm) was suspended horizontally at a depth of 1.5 m directly beneath a pontoon near to the seaward entrance of the marina. Panels were faced down towards the seabed in order to reduce light and sedimentation levels and therefore select for sessile fauna (rather than algae). Faunal assemblages were targeted because they are relatively well known (in terms of both taxonomy and species-specific physiology), often comprise multiple non-native species, and are relatively speciose. After a colonisation phase of ∼6 weeks, 30 panels were transported in cool boxes to the mesocosm facility at Plymouth Marine Laboratory (PML) within 1 h. Six panels were selected at random to remain on the grid throughout the experiment to serve as field-based controls. Seawater temperature at the study site was continuously monitored (every 20 min) with a ‘Hobo’ temperature logger attached to the experimental grids.

Within the mesocosm facility, 15 × 1 tonne capacity tanks were employed for Phase 2 (Fig. S1). Each tank held 0.58 m^3^ of freshly collected seawater which was constantly circulated using a peristaltic pump (Sci-Q 323; Walston Marlow Bredel, Falmouth, UK) and aerated by 2 air stones. Tanks were randomly assigned to one of 3 HW Magnitude treatments; 5 control tanks (hereafter ‘C’) were maintained at ambient sea temperature at the collection site (∼16.0 °C), 5 tanks were maintained at 3 °C above ambient (hereafter ‘T1’) and 5 tanks were maintained at 5 °C above ambient (hereafter ‘T2’). Temperatures in the control tanks were maintained through equilibration with air temperature within the mesocosm facility, which was controlled with a Refrigerating Heat Pump unit (Bartlett Refrigeration Limited, Exeter, UK). Temperatures in the treatment tanks were elevated and maintained using aquarium heaters (AquaVital 300W submersible heater thermostat) and electrical timers (Fig. 1). HW Magnitudes were representative of actual short-term seawater warming anomalies recorded in marine habitats (e.g., Jones, Berkelmans & Oliver, 1997; Garrabou et al., 2009; Wernberg et al., 2013). Two panels were selected at random and allocated to each tank, 1 for a 1 week HW duration and the other for a 2 week duration treatment (see below). Panels were held horizontally in the tanks, facing downwards, by attachment to a 1 m^2^ suspended plastic mesh.

Throughout the experimental phase, ‘Hobo’ loggers were deployed in each tank to record temperature every 15 min, and salinity and temperature were manually recorded 3 times per week and adjustments made as necessary. Approximately 0.2 tonnes of seawater per tank (i.e., 1/3 of the total volume) was replaced each week. Natural light regime was approximated using daylight simulation lights within the mesocosm with an average 12-h photoperiod per day. Throughout Phase 2, panel assemblages were fed to satiation (as indicated by continuous faecal production and replete ascidian intestines) through the addition of an algal cell suspension (800 ml per tank, three times per week). The suspension comprised a mix of the unicellular algae *Isochrysis galbana*, *Tetraselmis suecica* and *Rhinomonas reticulata* (cultured at PML) in seawater, with an average cell density of ∼5 × 10^6^.

Following a 1 week HW duration, 15 panels (i.e., 1 panel per tank) were returned to their original grid in Torquay Marina for a recovery period (Phase 3). After a further week, the remaining 15 panels were also returned to their original grid (Fig. 1). Following a recovery period of ∼3 weeks for the 1-week HW duration and ∼2 weeks for the 2-week HW duration panels, all panels were harvested and preserved in 80% IMS for subsequent scoring and analysis (see below). The entire 10-week experiment (Phase 1–3) was repeated 3 times, with each experimental HW ‘Timing’ commencing ∼2 weeks apart (from May 25th until June 21st), so that the timing of the simulated warming events was staggered throughout the summer (Fig. 1).

### Panel analysis

Panel assemblages were quantified at the end of Phase 1, 2 and 3. Panels were removed from the mesocosm tanks (Phase 1 and 2) or IMS containers (Phase 3) and submerged in a shallow Pyrex dish of seawater/IMS for examination under a dissecting light microscope. A wire mesh overlay, which projected 121 small squares (∼1.3 × 1.3 cm) for scoring, was used to analyse the inner 15 × 15 cm portion of each panel (the peripheral 2.5 cm was not scored to account for edge effects). Sessile organisms were identified to as high a taxonomic level as possible, always to genus and predominantly to species, and quantified by scoring every individual (solitary taxa) or colony (colonial taxa) occurring in each sub-sampled square of the scoring area. Any individual or colony occupying more than one square was scored multiple times accordingly, thereby generating an abundance score weighted by spatial coverage. Colonies or individuals that were heavily overgrown and clearly smothered by other encrusting taxa were assumed to be non-functional and were not counted. After the final analysis (Phase 3), all sessile fauna was scraped from the panel, dried at >60 °C for 48 h and then weighed to determine the total dry biomass of each panel.

### Statistical analysis

Data were analysed as 2 independent experiments based on HW duration (i.e., 1 and 2 week exposures), as direct comparisons were not appropriate due to potential confounding effects of total time spent in the mesocosms and in recovery (Phase 3). Instead, the effects of HW Magnitude were examined for each dataset separately before making qualitative comparisons of their relative effects between the 1 and 2 week HW durations. Initially, patterns in assemblage structure at the end of the experiment (i.e., after the Phase 3) were compared between the HW Magnitude treatments (i.e., control, T1, T2 and field controls) for each HW Timing (i.e., Timing 1, 2 and 3). A Bray-Curtis similarity matrix based on square-root transformed abundance data was used to construct Principal Coordinate Analysis (PCO) plots. Permutational multivariate analysis of variance (PERMANOVA) was used to examine differences between treatments, by employing a 2-way crossed design with HW Magnitude and Timing as fixed factors. Tests used 4,999 permutations under a reduced model, with significance accepted at *P* < 0.05. In addition, variability in assemblage level metrics (i.e., total abundance, species richness, total biomass) at the end of the experiment was examined with univariate permutational ANOVA (using the same model described above but with similarity matrices based on Euclidean distances).

As panel assemblage structure was highly variable after the colonisation stage (Phase 1), further analyses were conducted on *relative* changes in assemblage structure, in addition to *absolute* assemblage structure at the end of the experiment. Here, the percent cover of each taxa at the end of the colonisation phase (Phase 1) was subtracted from the final percentage cover recorded at the end of the experiment (Phase 3). Thus, a positive value indicated an increase in spatial cover from the initial colonisation phase through the experimental and grow-out phase whereas a negative value indicated a decrease. Multivariate assemblage-level analysis were then conducted on relative changes, first by adding 1,000 to each score to generate positive values, then by constructing similarity matrices based on Euclidean distances of untransformed data. For the 1 week and 2 week HW duration experiments independently, differences in assemblage structure between HW Magnitude and Timing were then visually and statistically examined using the same approach described above for patterns of absolute assemblage structure. Where a significant interaction between HW Magnitude and Timing was detected, pairwise comparisons within each level of the interaction term were conducted. Patterns of relative change in the abundance of dominant species, both native and non-native, in response to the HW Magnitude treatments were also examined, using one-way PERMANOVA to test for differences between HW Magnitude within each Timing. All statistical procedures were conducted with the PERMANOVA add-on (Anderson, Gorley & Clarke, 2008) for PRIMER v.6 software (Clarke & Warwick, 2001).

## Results

### Heat wave simulations

Temperatures in the treatment tanks were stable throughout the experimental period (Phase 2), with mean values (±SD) of 16.9 ± 0.5 °C, 19.8 ± 0.3 °C and 21.8 ± 0.4 °C for C, T1 and T2, respectively. Control tank temperatures were well-aligned with monthly mean temperature for the study site (based on *in situ* logger data for 2010–2013). Daily mean temperatures for the field site indicated that a short-term warming event occurred through mid-July 2013 (Fig. 1). Even so, daily mean temperatures for T1 and T2 were greater than the maximum daily mean recorded in the field (Fig. 1).

### Assemblage level responses

A total of 34 taxa (24 species and 10 distinct genera), 7 of which were classified as non-native, were recorded on the 108 experimental panels (Table S1). Overall, panel assemblages were well developed by the commencement of the experimental stage (Phase 2), with an average richness of 14.5 ± 0.5 species per panel and an average total faunal cover of 67.4 ± 3.5% (Fig. S2). At the end of the experimental runs (Phase 3) average panel richness and total cover (%) was 15.4 ± 0.3 and 80.3 ± 6.7%, respectively (Fig. S2). Following colonisation (Phase 1), panel assemblages were fairly variable in structure, most likely due to high spatial variability in settlement and recruitment (Fig. S3). Even so, panel assemblages within each experimental event were generally clustered together whereas no partitioning between the randomly assigned treatments was observable (Fig. S3).

**Figure 2:**
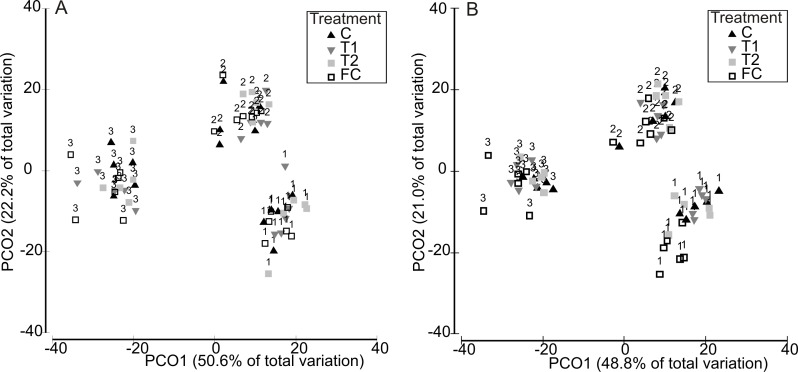
Multivariate assemblage structure following the recovery period (Phase 3). Principal Coordinates Analysis (PCO) plots indicating absolute multivariate assemblage structure at the end of the experiment, following recovery phase (Phase 3) for the 1 week (A) and 2 week (B) HW durations. Multivariate partitioning is based on Bray-Curtis similarities of square root transformed abundance data. Centroid symbols indicate the HW Magnitude treatment for each panel (C = Controls at ambient temperature, T1 = +3 °C, T2 = +5 °C) and labels indicate the different HW Timings (i.e., experimental runs).

**Figure 3:**
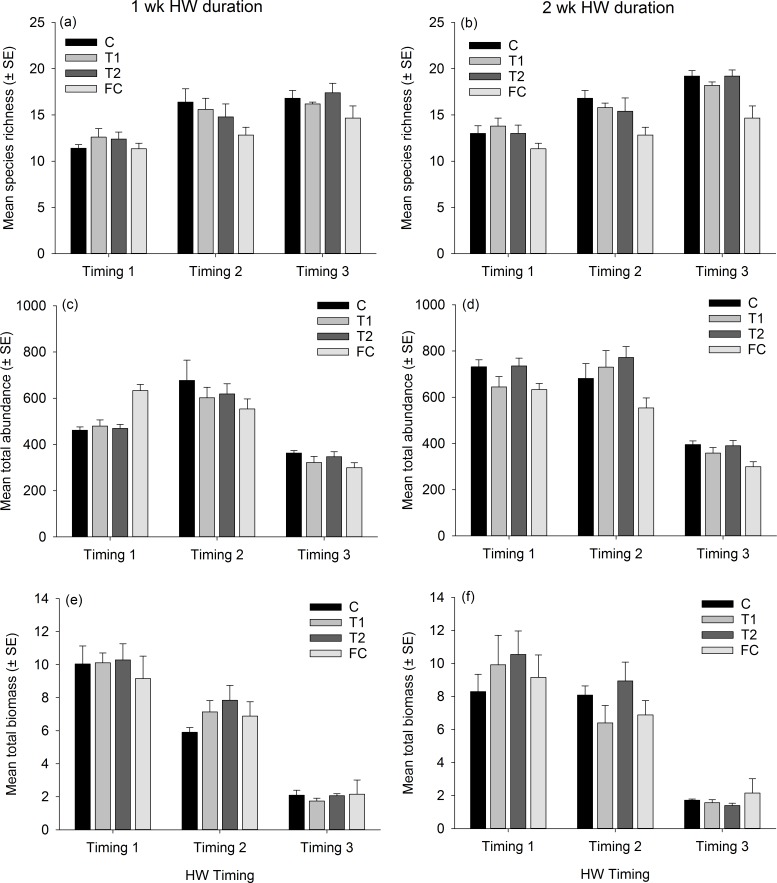
Assemblage-level metrics following the recovery period (Phase 3). Mean (±SE) species richness (A, B), total abundance (C, D) and total dry weight biomass (E, F; in g) of panel assemblages following the recovery phase (Phase 3) for the 1 wk HW duration (A, C, E) and 2 wk HW duration (B, D, F) experiments. Magnitude treatments, are: C = Controls at ambient temperature, T1 = +3 °C and T2 = +5 °C, which were run for either 1 or 2 wk durations. Assemblage data from Field Controls (FC) are also shown.

Partitioning in *absolute* multivariate assemblage structure at the end of the experiment was principally related to the timing of the experimental run, in that panel assemblages were clustered by the ‘HW ‘Timing’ rather than by HW ‘Magnitude’ for both the 1 week and 2 week durations (Fig. 2). Within each Timing, there was no clear partitioning in multivariate assemblage structure between the Magnitude treatments (i.e., C, T1, T2), indicating that the HW simulations had little effect on overall assemblage structure based on absolute abundances. However, the assemblages on the field controls (FC) were generally dissimilar to those on the experimental panels (Table 1 and Fig. S4), suggesting that the 1 or 2 weeks spent in the mesocosm facility influenced community succession to some degree, as could be expected. Even so, when compared to variability between Timings, differences in assemblage structure between field controls and experimental controls were minimal (Fig. 2). For the 1 week HW duration, a significant effect of Magnitude was detected, and post-hoc tests indicated that the C panels supported distinct assemblages from the T2 panels (Table 1). For the 2 week HW duration, significant differences between assemblages on C panels and those on T1 and T2 panels was recorded following HW Timing 2 (Table 1). Univariate permutational ANOVA indicated that total abundance and species richness differed between treatments, but only following the 2 week duration experiments (Table S2). Pairwise tests showed that the Field Controls differed from the experimental treatments (which were all statistically similar), again suggesting some effect of the experimental procedure. Plots of species richness, total abundance and total biomass (dry weight) at the end of the experiment (after Phase 3) showed no clear differences between the HW Magnitude treatments (Fig. 3).

**Table 1:** PERMANOVAs to test for effects of HW simulations on absolute assemblage structure. PERMANOVAs to test for differences in multivariate assemblage structure (after Phase 3) between HW Timings and HW Magnitudes based on absolute abundance data. Permutations (4,999 under a reduced model) were based on Bray-Curtis similarity matrices constructed from square-root transformed abundance data. Main tests were conducted on both the 1-week (A) and 2-week (B) HW duration experiments separately. Significant *P* values (at *P* < 0.05) are shown in bold. Significant Magnitude/Magnitude × Timing interaction effects were investigated further with post-hoc pairwise comparisons.

Source	df	SS	F	*P*
**(A)**				
Magnitude	3	1,226	1.88	**0.010**
Timing	2	2,7267	62.87	**0.001**
Ma × Ti	6	1,313	1.01	0.485
Residual	52	11,276		
Total	63	41,467		
*Post-hoc tests for Magnitude*: FC ≠ C & T2, C ≠ T2				
**(B)**				
Magnitude	3	2,420	4.25	**0.001**
Timing	2	25,517	67.20	**0.001**
Ma × Ti	6	2,101	1.85	**0.001**
Residual	52	9,872		
Total	63	40,312		
*Post-hoc tests for Ma* × *Ti:*				
Timing 1: FC ≠ C & T1 & T2				
Timing 2: FC ≠ C & T1 & T2, C ≠ T1 & T2				
Timing 3: FC ≠ C & T1 & T2				

**Figure 4:**
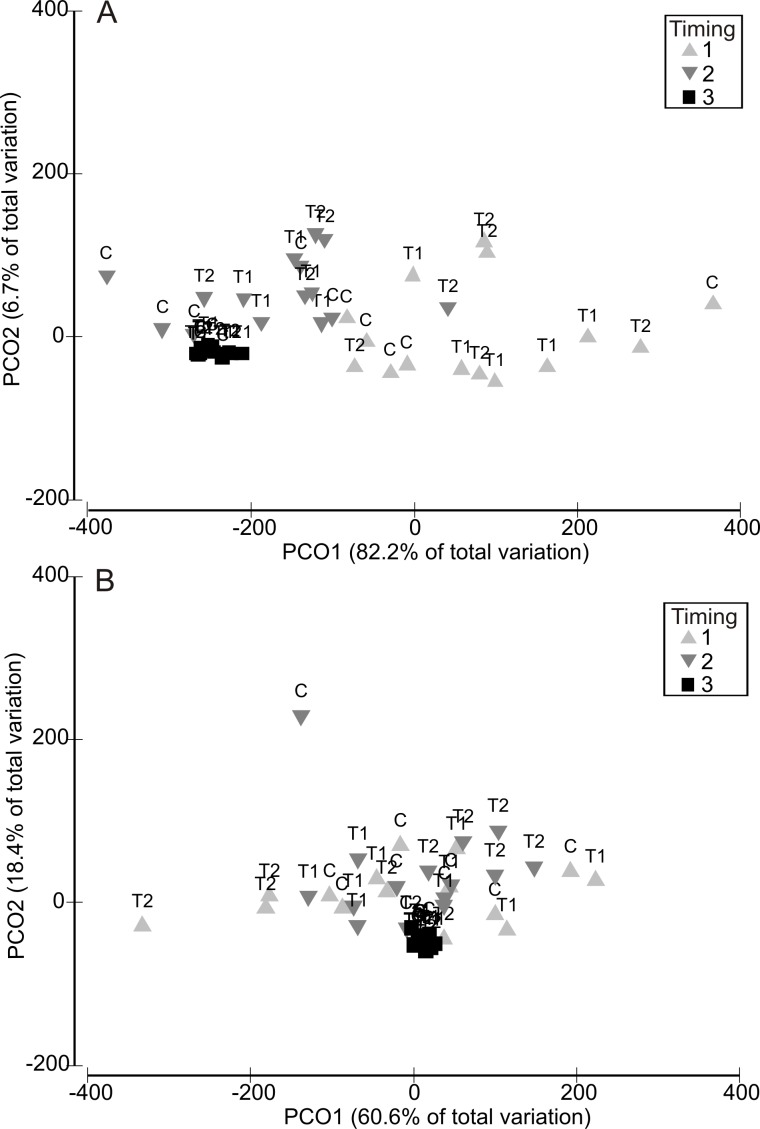
Relative changes in assemblage structure following the recovery period (Phase 3). Principal Coordinates Analysis (PCO) plots indicating relative changes in multivariate assemblage structure between the colonisation phase (Phase 1) and the recovery phase (Phase 3) for the 1 week (A) and 2 week (B) HW exposures. Multivariate partitioning is based on Euclidian distances between untransformed differences in abundance between Phase 1 and Phase 3. Centroid symbols indicate the HW Magnitude treatment for each panel (C = Controls at ambient temperature, T1 = +3 °C, T2 = +5 °C) and labels indicate the different HW Timings (i.e., experimental runs).

**Figure 5:**
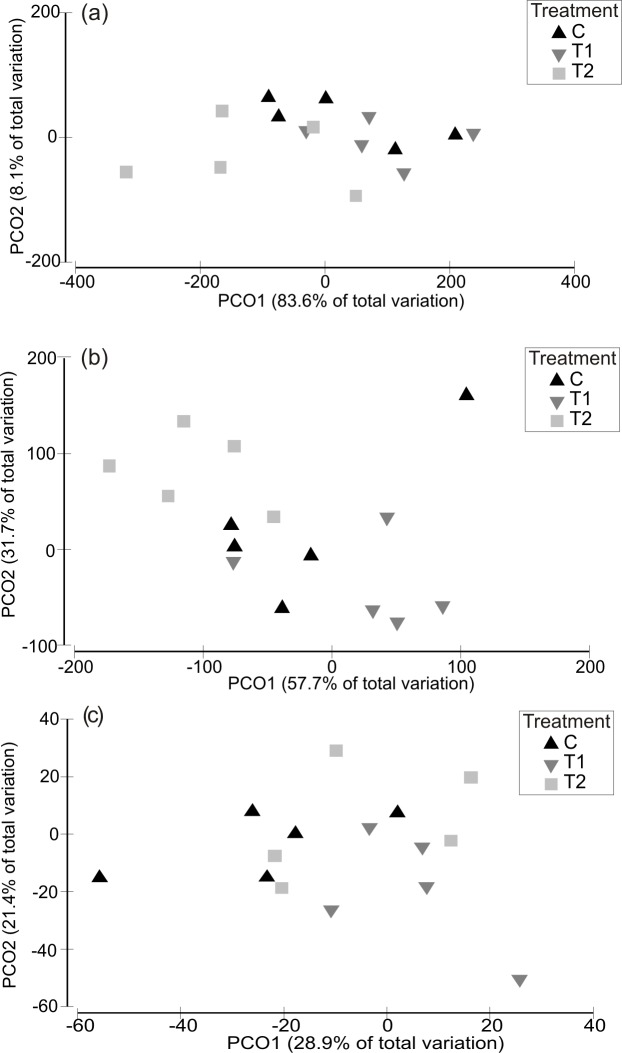
Relative changes in assemblage structure following 2-week HW duration treatments, shown separately for each HW Timing. Principal Coordinates Analysis (PCO) plots indicating relative changes in multivariate assemblage structure between the colonisation phase (Phase 1) and the recovery phase (Phase 3) for HW Timing 1 (A), 2 (B) and 3 (C) individually, following a 2 week HW duration. Multivariate partitioning is based on Euclidian distances between untransformed differences in abundance between Phase 1 and Phase 3. Centroid symbols indicate the HW Magnitude for each panel (C = Controls at ambient temperature, T1 = +3 °C, T2 = +5 °C).

Analysis of variability in assemblage structure based on *relative* changes in abundance (i.e., the difference between initial abundances after Phase 1 and final abundances after Phase 3) showed that responses to the HW Magnitude treatments varied considerably between HW Timings (Fig. 4). For example, variability in shifts in assemblage structure was much greater for Timing 1 and 2 compared with Timing 3, for both the 1 and 2 week HW durations (Fig. 4). PERMANOVA detected significant differences in assemblage structure (based on relative changes in abundance) between Timings for both HW durations (Table 2). For the 1 week HW duration experiments, the main and interactive effects of Magnitude were non-significant (Table 2). However, for the 2 week HW duration experiment, a significant Magnitude × Timing interaction was detected (Table 2). Post-hoc pairwise comparisons between HW Magnitudes for each HW Timing showed that assemblage structure for the T2 panels was significantly different to those observed for the C and T1 panels following Timing 1 (Table 2). This was also evident in the PCO plots, with clear partitioning between T2 assemblages and C/T1 assemblages following Timing 1 (Fig. 5). For Timing 2 and 3, however, no significant differences were detected between HW Magnitudes (Table 2), although some partitioning between T2 and C/T1 assemblages was again evident following Timing 2 (Fig. 5).

**Table 2:** PERMANOVAs to test for effects of HW simulations on relative changes in assemblage structure. PERMANOVAs to test for differences in multivariate assemblage structure between HW Magnitudes and Timings, based on relative changes in species abundances (change in abundance between Phase 1 and Phase 3). Permutations (4,999 under a reduced model) were based on Euclidean distances between untransformed changes in abundance. Main tests were conducted on both the 1-week (A) and 2-week (B) HW duration experiments separately. Significant *P* values (at *P* < 0.05) are shown in bold. Significant Magnitude/Magnitude × Timing interaction effects were investigated further with post-hoc pairwise comparisons.

Source	df	SS	F	*P*
**(A)**				
Magnitude	2	32,932	1.96	0.100
Timing	2	536,120	31.93	**0.001**
Ma × Ti	4	21,862	0.65	0.753
Residual	36	302,220		
Total	44	893,130		
**(B)**				
Magnitude	2	11,938	0.57	0.737
Timing	2	112,350	5.43	**0.001**
Ma × Ti	4	193,060	4.66	**0.002**
Residual	36	372,660		
Total	44	690,010		
*Post-hoc tests for Ma* × *Ti:*				
Timing 1: C = T1 ≠ T2				
Timing 2: C = T1 = T2				
Timing 3: C = T1 = T2				

### Species level responses

**Figure 6:**
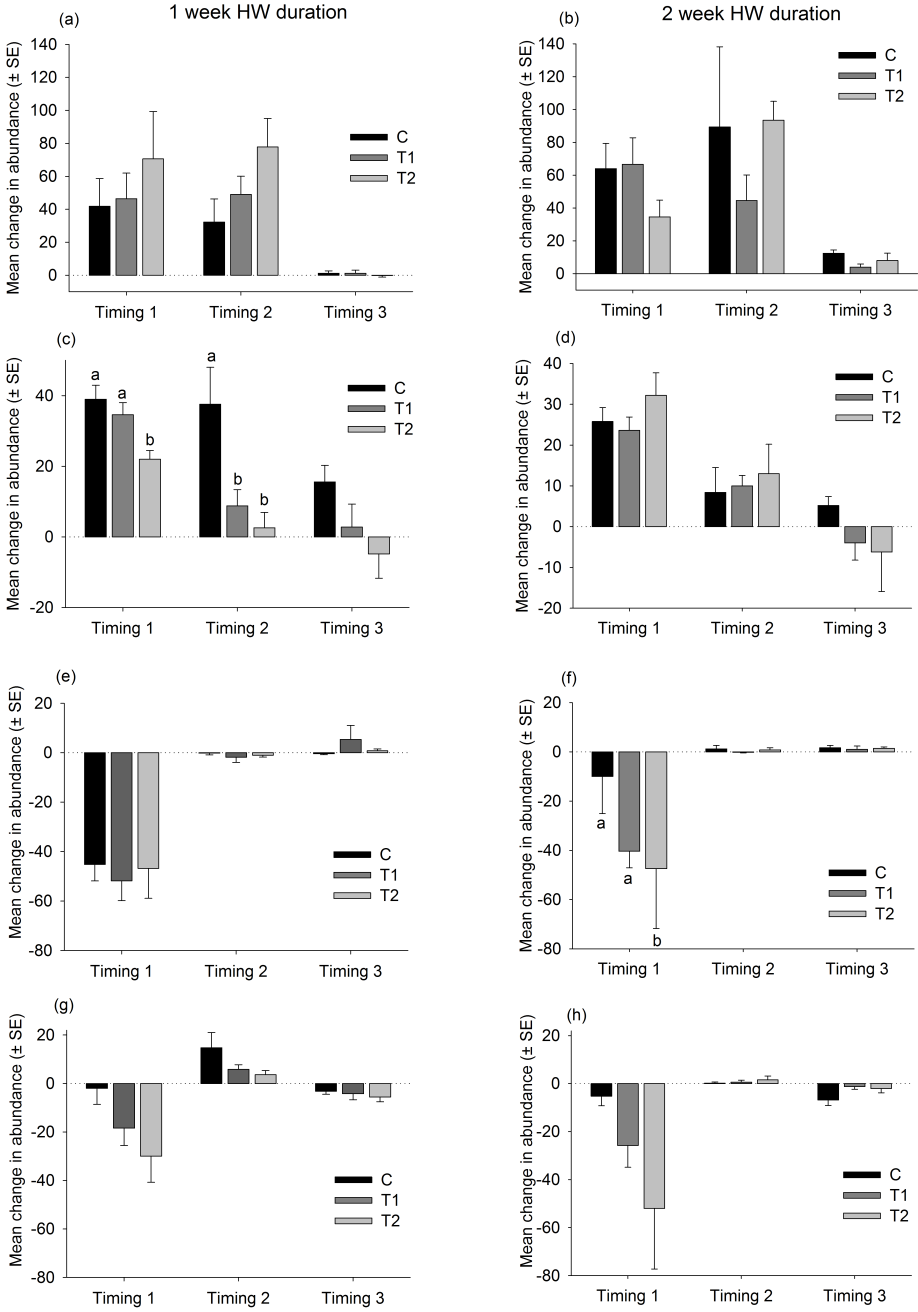
Relative changes in abundances of native species following HW simulations. Responses of dominant native species to the HW Magnitude treatments (C, black bars; T1, dark grey bars; T2, light grey bars) after 1 week (A, C, E, G) and 2 week (B, D, F, H) HW durations. The average change in abundance (between Phase 1 and 3, *n* = 5 panels, ±SE) is shown for *Ciona intestinalis* (A, B), *Botryllus schlosseri* (C, D), *Electra pilosa* (E, F) and *Balanus crenatus* (G, H). Lower case letters indicate significant differences between HW magnitudes (at *P* < 0.05, based on one-way PERMANOVAs within each HW Timing and duration).

There were no consistent trends in the responses of native dominant species to the HW Magnitude treatments (Fig. 6). Relative changes in abundances were variable, both between species and between Timings for the same species (Fig. 6). The only significant differences between HW Magnitudes were observed for the colonial ascidian *Botryllus schlosseri*, after 2 of the 1-week HW duration events, and the bryozoan *Electra pilosa*, after a 2-week HW event (Fig. 6 and Table S3). For *B. schlosseri*, the rate of increase in abundance was significantly lower on the T2 panels compared with the control panels (Fig. 6 and Table S3). For *E. pilosa*, which decreased in abundance during the HW Timing 1, the rate of decline was greater on the T2 panels compared with the control panels (Fig. 6 and Table S3).

**Figure 7:**
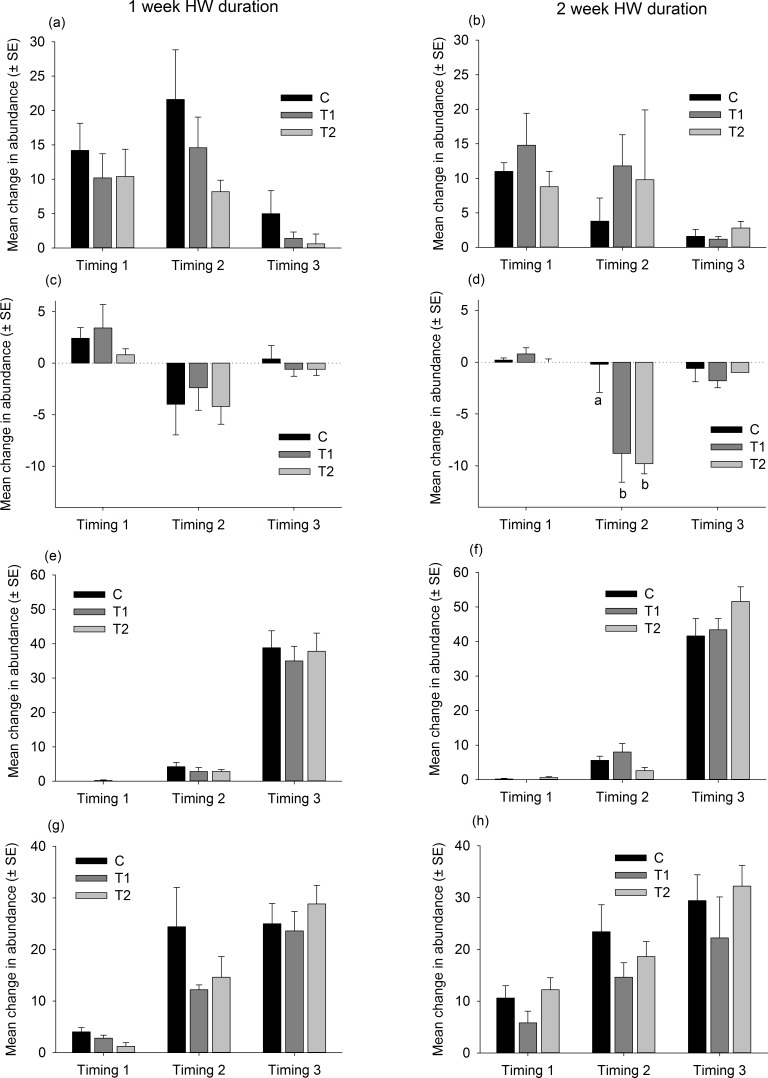
Relative changes in abundances of non-native species follwoing HW simulations. Responses of the most abundant non-native species to the HW Magnitude treatments (C, black bars; T1 dark grey bars, T2, light grey bars) after 1 week (A, C, E, G) and 2 week (B, D, F, H) HW durations. The average change in abundance (between Phase 1 and 3, *n* = 5 panels, ±SE) is shown for *Corella eumyota* (A, B), *Asterocarpa humilis* (C, D), *Bugula neritina* (E, F) and *Tricellaria inopinata* (G, H). Lower case letters indicate significant differences between HW Magnitudes (at *P* < 0.05, based on one-way PERMANOVAs within each HW Timing and duration).

The responses of non-native species were similarly variable and no clear trends were observed (Fig. 7 and Table S4). The ascidian *Corella eumyota* and the bryozoans *Bugula neritina* and *Tricellaria inopinata* generally increased in abundance between Phase 1 and Phase 2 and the magnitude of increase did not vary between the HW Magnitude treatments (Fig. 7 and Table S4). The compass ascidian, *Asterocarpa humilis*, decreased in abundance in HW Timing 2, and the magnitude of decline was significantly greater on the HW Magnitude treatments T1 and T2 compared with the control panels (Fig. 7 and Table S4).

## Discussion

Contrary to our expectations, the simulated HW events had no clear, consistent impacts on the abundance of species or the structure of sessile assemblages. The first hypothesis, that long duration high magnitude HWs would alter assemblage structure was supported to some degree, as assemblages subjected to +5 °C for 2 weeks were distinct to those held at ambient temperature or at +3 °C (as determined by relative changes in species’ abundances). However, this pattern was observed for only 1 of the 3 HW Timings. The second hypothesis, that non-native species would be less impacted by warming events than native species, was not supported as there were no clear trends in responses of non-native species to simulated HWs. The third hypothesis, which stated that changes in species abundances and shifts in community structure would be consistent between HW Timings, was also not supported.

The support for Hypothesis 1 emerged from the first HW Timing, as the T2 assemblages were dissimilar to the C and T1 assemblages, based on relative changes in species abundances. The observed differences in assemblage structure were principally driven by a greater decline in the abundance of the bryozoan *Electra pilosa*, the ascidian *Ciona intestinalis* (non-significant) and the barnacle *Balanus crenatus* (non-significant) on panels subjected to the high-magnitude HW treatment (T2). These species are all widespread in cold temperate seas, and there is some evidence to suggest that their ecological performance (i.e., filtration rates, growth) declines at temperatures in excess of ∼21 °C (Southward, 1964; Menon, 1972; Peterson & Riisgard, 1992). As such, it is likely that the highest-magnitude warming treatment negatively impacted their performance and resulted in lower abundances compared with the cooler treatments. What is not clear, however, is why these patterns were not consistently observed across the HW Timings. Interestingly, the colonial ascidian *Botryllus schlosseri* was negatively impacted by the warmer treatments in the short duration experiment, but no significant differences were observed in the longer duration experiment. *B. schlosseri*, which is widespread outside its native range, has a broad thermal tolerance and temperatures in excess of 23 °C do not inhibit growth or survival (Epelbaum et al., 2009; McCarthy, Osman & Whitlatch, 2007). As such, it could be that short-term elevated temperatures had an indirect effect, through altered competitive interactions for example, but this requires further investigation.

Our results do not align with other studies on marine community-level responses to short-term warming events, which have reported high levels of species mortality, decreases in diversity and significant shifts in community structure (Allison, 2004; Sorte, Fuller & Bracken, 2010; Smale et al., 2011; Eggers, Eriksson & Matthiessen, 2012; Smale & Wernberg, 2012). We suggest the reasons for this divergence are two-fold. Firstly, several of these previous studies (Allison, 2004; Sorte, Fuller & Bracken, 2010; Eggers, Eriksson & Matthiessen, 2012) subjected assemblages to heat stress events in the magnitude of ≥10 °C above ambient temperature. As such, the lack of ecological responses observed in the current study is likely to be a consequence of a lower-intensity HW Magnitude treatment. Given that warming anomalies of +2–4 °C above long-term average temperatures are typical of the most extreme marine HWs that occur in nature (Garrabou et al., 2009; Wernberg et al., 2013), the magnitudes of warming treatments applied in the current study were more realistic. High intensity stress experiments are undoubtedly useful for examining physiological response mechanisms, identifying ecological winners and losers and predicting ‘worst case scenarios.’ We would argue, however, that such experiments may overestimate the effects of warming and downplay the resilience of populations and communities to temperature variability characteristic of natural systems (see Wernberg, Smale & Thomsen, 2012 for further discussion). Secondly, previous community-level warming studies that used heated settlement panels (Smale et al., 2011; Smale & Wernberg, 2012), examined the response of early-stage assemblages to short-term warming events, as opposed to the mature, fully developed assemblages studies here. Previous research would suggest that early life stages are the most susceptible to environmental stressors such as warming (see Byrne & Przeslawski, 2013; Harvey, Gwynn-Jones & Moore, 2013 for recent syntheses), and there is emerging evidence to suggest that the microbial processes which influence invertebrate settlement (i.e., biofilm development) are strongly affected by temperature (Russell et al., 2013; Whalan & Webster, 2014). It seems likely, therefore, that early successional stages comprising recently-settled organisms are more sensitive to short-term warming events than developed assemblages comprising mature stage organisms (but see Sorte, Fuller & Bracken, 2010), but further work is required.

Our study also demonstrates the importance of assemblage type and biogeographical context in determining ecological responses to short-term warming events. The sessile invertebrate species forming the model assemblages used in the present study are generally widespread in temperate ecosystems and have wide environmental tolerances, being common in marinas and other habitats characterised by large fluctuations in sediments, salinity and temperature. The majority of the species sampled are also found further south, in warmer waters. This could explain the observed resilience of the assemblages to realistic HW Magnitude treatments, which perhaps did not exceed the thermal thresholds of these populations. Indeed, many species exhibited a positive response to warming, in that they increased in abundance throughout the experiment even when subjected to the highest-magnitude warming treatment. Recent work by Lord & Whitlatch (in press) has experimentally shown that ecological responses to temperature of sessile invertebrates varies with latitude, as populations at the ‘warm edge’ of species ranges are negatively impacted by warming. In the current study, the species recorded were not sampled from range edge populations and, as such, demonstrated capacity to tolerate a simulated HW of +5 °C for 2 weeks. Clearly, populations found at the range edge of species’ distributions are the most vulnerable to extreme warming events, as has been shown in recent marine HWs in both Europe (Garrabou et al., 2009; Puce et al., 2009) and Australia (Smale & Wernberg, 2013; Wernberg et al., 2013).

Our results did not support our second hypothesis, that short-term warming events would be less detrimental, or even favourable, to non-native species compared with native species. The only significant change in abundance we observed was for the non-native compass ascidian *Asterocarpa humilis*, which exhibited greater declines in abundance at higher temperatures. This is somewhat contradictory to previous work, which shown that warming may favour non-native species and facilitate their spread (Stachowicz et al., 2002; Sorte, Williams & Zerebecki, 2010; Smale et al., 2011; Sorte & Stachowicz, 2011). Again, this is likely to be context-dependent, as the non-native species sampled in the current study were seemingly not at a competitive advantage at higher temperatures, probably because native species generally remained within their thermal thresholds even at the high-magnitude treatment. Even so, there is widespread consensus that longer-term gradual warming will facilitate the spread of non-native species in North Atlantic marine ecosystems (Occhipinti-Ambrogi, 2007; Pederson et al., 2011).

With regards to the realism of our HW manipulations, it is important to note that many naturally-occurring marine HWs are driven by oceanographic processes that cause the influx of warm water (and associated dispersal stages of warm-water species) into cooler zones (e.g., Wernberg et al., 2013). As such, ‘real world’ HWs can cause a reshuffling of the species pool through the advection of warm water species, which may be competitively superior to the cool water species during (and after) the warm water event. Our experiments did not involve a reshuffling of the species pool, which could include the addition of warm water non-native species for example, and therefore did not incorporate all possible mechanisms of ecological change. However, marine HWs such as the Mediterranean event of 2003 (Garrabou et al., 2009) are characterised by *in situ* warming driven by anomalous air-sea heat transfer, which has a direct physiological effect of the local species pool. As such, our HW manipulations are more akin to this type of event.

The greatest source of variability we observed was related to the timing of substrate colonisation and assemblage development. It has long been known that sessile marine fauna exhibit pronounced variability in their timings of reproduction, recruitment and growth (e.g., Thorson, 1950) and, as a result, the timing of the commencement of succession processes can strongly influence assemblage structure (Harms & Anger, 1983). Here, the onset of colonisation was staggered by just 2 weeks between experiments, yet temporal variability in assemblage structure far outweighed that caused by the warming treatments. Moreover, the HW Magnitude treatments had discernible effects on sessile assemblage structure following only one of the three HW Timings, which implied that some assemblage types are more susceptible to warming events than others. Interestingly, the ‘Timing 1’assemblages, which were most impacted by the simulated HWs, generally had lower species richness and greater total abundance values compared with the subsequent assemblages subjected to Timing 2 and 3. This corresponds with the findings of Allison (2004), which manipulated species diversity and thermal stress on rocky shores to show that community responses are dependent on the characteristics of both the stress and the species present in the community, which varies in time. Clearly, further work on the influence of diversity on community resistance (and resilience) to marine HWs is needed to better understand ecological responses to temperature variability.

## Conclusions

Our realistic HW simulations had few discernible effects on the diversity, structure and successional trajectories of sessile invertebrate assemblages, which were resistant to short-term warming. The study highlights the importance of (i) conducting experiments on naturally-occurring suites of species, at observed densities, (ii) simulating naturally-occurring stress events though realistic treatments and (iii) considering biogeographical and temporal context in predictions of ecological responses to warming. Such an approach will improve realism and expand the inference space of climate change experiments, which is necessary to develop useful predictions of ecological responses to future change.

## Supplemental Information

10.7717/peerj.863/supp-1 Figure S1 Experimental approach Preparing the panels for initial deployment at the Torquay Marina study site (A) and the mesocosm facility at Plymouth Marine Laboratory (B).

10.7717/peerj.863/supp-2 Figure S2 Typical panel assemblages following Phase 1 and Phase 3 Representative panel assemblages following: (A) the initial colonisation period (Phase 1) and (B) the recovery period (Phase 3). Panels (20 × 20 cm in size) were colonised by a range of marine invertebrates, including colonial ascidians (e.g., *Botryllus schlosseri*), solitary ascidians (e.g., *Ascidiella aspersa*), cheilostome bryozoans (e.g., *Tricellaria inopinata* and *Electra pilosa*) and calcareous polychaetes (e.g., *Spirobranchus* sp.).

10.7717/peerj.863/supp-3 Figure S3 Multivariate assemblage structure follwoing the colonisation period (Phase 1) Principal Coordinates Analysis (PCO) plots indicating multivariate assemblage structure at the end of the colonisation period (Phase 1) prior to the experimental period (Phase 2). Multivariate partitioning is based on Bray-Curtis similarities of square root transformed abundance data. Centroid symbols indicate the different HW Timings (i.e., experimental runs) and labels indicate the randomly assigned HW Magnitude treatment for each panel (C = Controls at ambient temperature, T1 = +3 °C, T2 = +5 °C). Panels selected for the 1-week HW duration (A) and the 2 week HW duration (B) are shown separately.

10.7717/peerj.863/supp-4 Figure S4 Multivariate assemblage structure following the recovery period (Phase 3), shown separately for each HW duration and Timing Principal Coordinates Analysis (PCO) plots indicating multivariate assemblage structure at the end of the experiment (following ‘recovery’ Phase 3). Multivariate partitioning is based on Bray-Curtis similarities of square root transformed abundance data. Assemblage structure for each HW Magnitude treatment (C = Controls at ambient temperature, T1 = +3 °C, T2 = +5 °C, FC = Field Control) is shown for 1 week HW durations for each HW Timing (A–C) and 2 week HW durations for each Timing (D–F) separately.

10.7717/peerj.863/supp-5 Table S1 Species list List of species recorded on panels, with NIS status in the UK, life history strategy (colonial versus solitary) and occurrence of each species (i.e., % of panels on which species was recorded) also shown.

10.7717/peerj.863/supp-6 Table S2 Variability in assemblage-level metrics following the recovery period (Phase 3) Univariate permutational ANOVAs to test for differences in assemblage level metrics between HW Timings and Magnitudes, based on final values of total abundance (a,b), species richness (c,d) and dry weight biomass (e,f). Permutations (4999 under a reduced model) were based on Euclidean distances between untransformed data. Main tests were conducted on both the 1-week (a,c,e) and 2-week (b,d,f) HW duration experiments separately. Significant *P* values (at *P* < 0.05) are shown in bold. Significant HW Magnitude effects were investigated further with post-hoc pairwise comparisons.

10.7717/peerj.863/supp-7 Table S3 Responses to native species to the HW simulations One-way univariate permutational ANOVAs to test for the effects of HW Magnitude on the abundance of dominant native species. Abundance values reflected the relative change in actual abundance between the colonisation period (Phase 1) and the end of the experiment (Phase 3), and are shown graphically in Fig. 5. Permutations (999 unrestricted) were based on Euclidean distances between untransformed change in abundance (plus 1,000). Significant *P* values (at *P* < 0.05) are shown in bold.

10.7717/peerj.863/supp-8 Table S4 Responses of non-native species to the HW simulations One-way univariate permutational ANOVAs to test for the effects of HW Magnitude on the abundance of non-native species. Abundance values reflected the relative change in actual abundance between the colonisation period (Phase 1) and the end of the experiment (Phase 3), and are shown graphically in Fig. 5. Permutations (999 unrestricted) were based on Euclidean distances between untransformed change in abundance (plus 1,000). Significant *P* values (at *P* < 0.05) are shown in bold.

10.7717/peerj.863/supp-9 Supplemental Information 9 Raw data Abundances of taxa recorded on each sample.
