# Supplementary figures and images for: Disentangling the impacts of heat wave magnitude, duration and timing on the structure and diversity of sessile marine assemblages
Source: PeerJ. 2015 Mar 26;3:e863. doi: 10.7717/peerj.863 (PMC4380158; doi:10.7717/peerj.863)

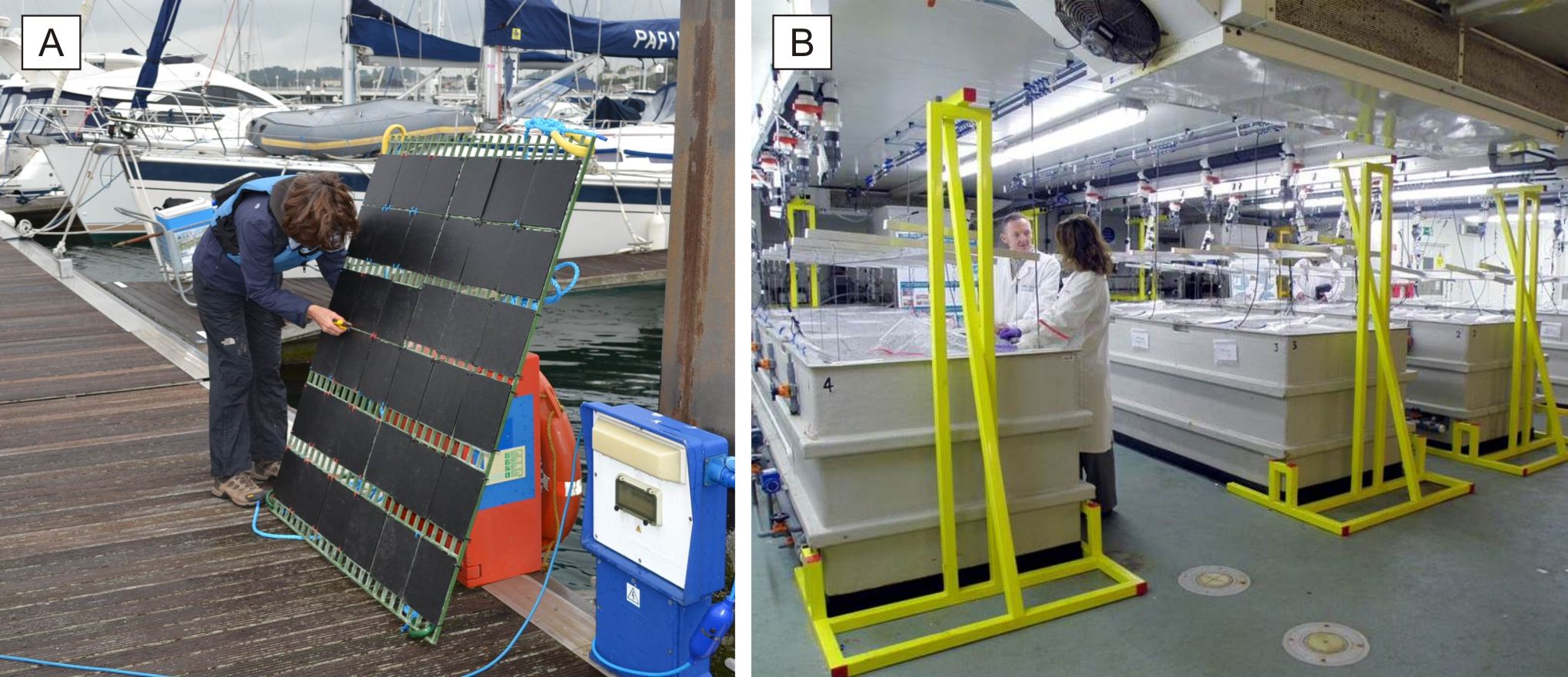

Supplement: Figure S1 — Preparing the panels for initial deployment at the Torquay Marina study site (A) and the mesocosm facility at Plymouth Marine Laboratory (B). [file peerj-03-863-s001.jpg]

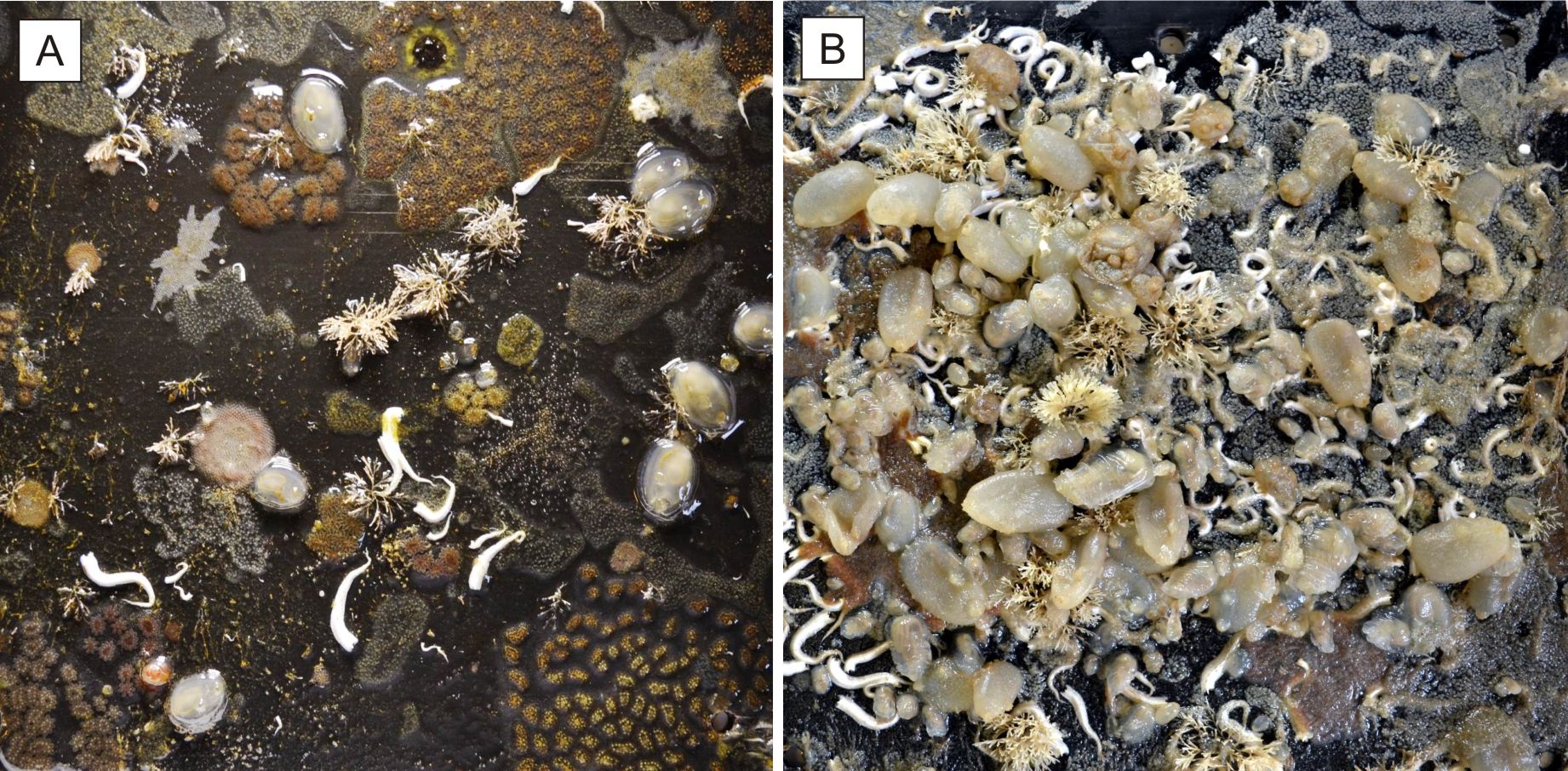

Supplement: Figure S2 — Representative panel assemblages following: (A) the initial colonisation period (Phase 1) and (B) the recovery period (Phase 3). Panels (20 × 20 cm in size) were colonised by a range of marine invertebrates, including colonial ascidians (e.g., Botryllus schlosseri), solitary ascidians (e.g., Ascidiella aspersa), cheilostome bryozoans (e.g., Tricellaria inopinata and Electra pilosa) and calcareous polychaetes (e.g., Spirobranchus sp.). [file peerj-03-863-s002.jpg]

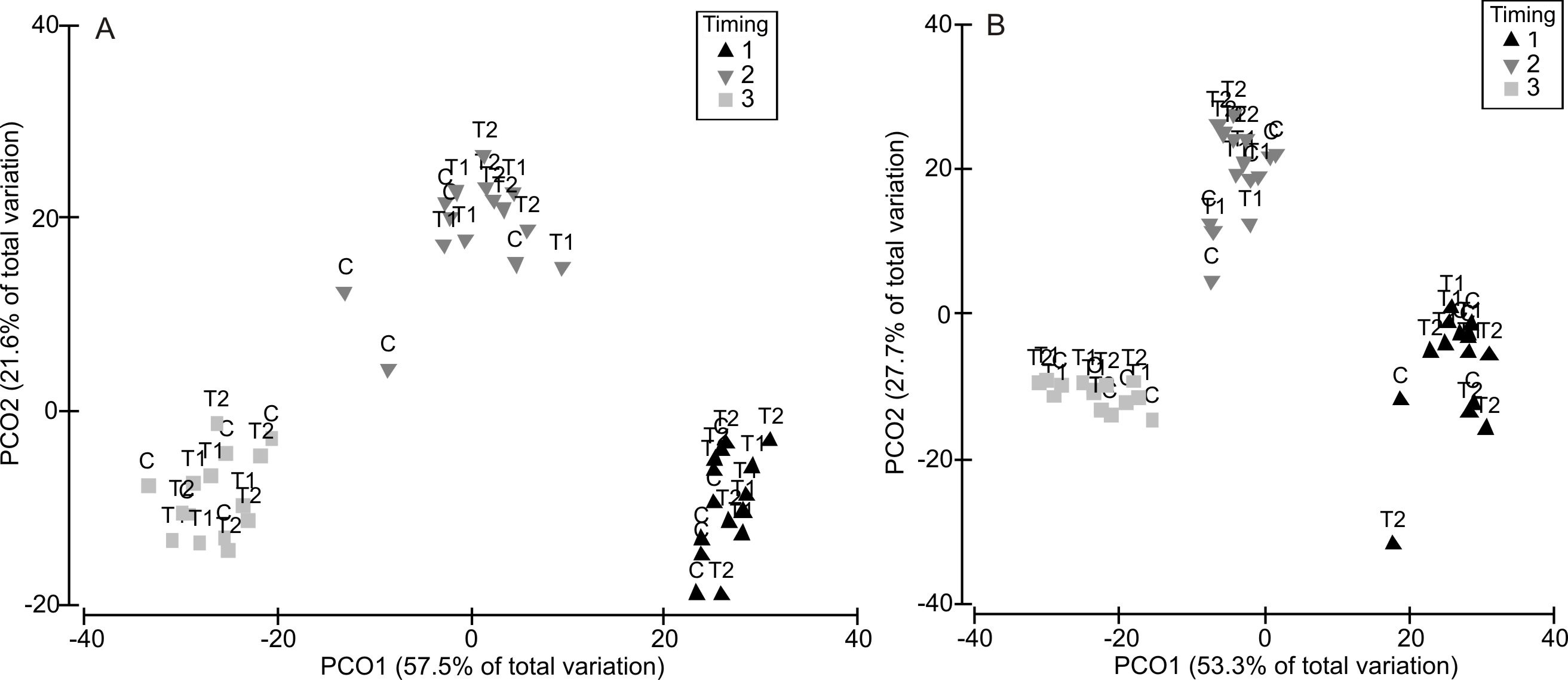

Supplement: Figure S3 — Principal Coordinates Analysis (PCO) plots indicating multivariate assemblage structure at the end of the colonisation period (Phase 1) prior to the experimental period (Phase 2). Multivariate partitioning is based on Bray-Curtis similarities of square root transformed abundance data. Centroid symbols indicate the different HW Timings (i.e., experimental runs) and labels indicate the randomly assigned HW Magnitude treatment for each panel (C = Controls at ambient temperature, T1 = +3 °C, T2 = +5 °C). Panels selected for the 1-week HW duration (A) and the 2 week HW duration (B) are shown separately. [file peerj-03-863-s003.jpg]

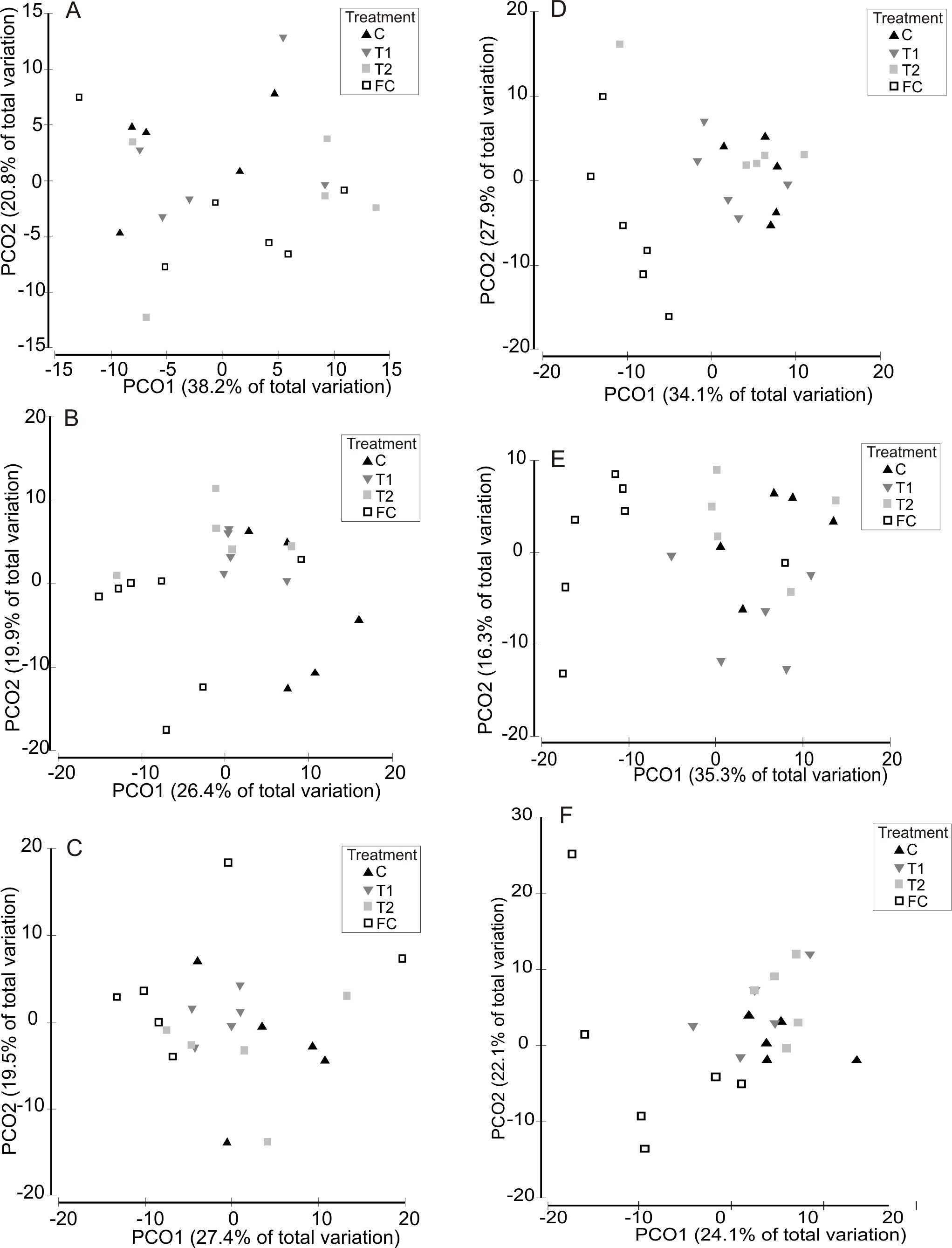

Supplement: Figure S4 — Principal Coordinates Analysis (PCO) plots indicating multivariate assemblage structure at the end of the experiment (following ‘recovery’ Phase 3). Multivariate partitioning is based on Bray-Curtis similarities of square root transformed abundance data. Assemblage structure for each HW Magnitude treatment (C = Controls at ambient temperature, T1 = +3 °C, T2 = +5 °C, FC = Field Control) is shown for 1 week HW durations for each HW Timing (A–C) and 2 week HW durations for each Timing (D–F) separately. [file peerj-03-863-s004.jpg]
