# Supplementary material for: Disentangling the impacts of heat wave magnitude, duration and timing on the structure and diversity of sessile marine assemblages
Source: PeerJ. 2015 Mar 26;3:e863. doi: 10.7717/peerj.863 (PMC4380158; doi:10.7717/peerj.863)
Supplement: Table S1 — List of species recorded on panels, with NIS status in the UK, life history strategy (colonial versus solitary) and occurrence of each species (i.e., % of panels on which species was recorded) also shown. [file peerj-03-863-s005.pdf]

Table S1. List of species recorded on panels, with NIS status in the UK, life history strategy (colonial versus solitary) and occurrence of each species (i.e. % of panels on which species was recorded) also shown.

Taxon	Species	Non-native?	Colonial?	Occurrence (%)
Ascidians	*Aplidium glabrum*	N	Y	1
	Ascidiella aspersa	N	N	98
	Asterocarpa humilis	Y	N	60
	Botrylloides leachii	N	Y	1
	Botryllus schlosseri	N	Y	100
	Ciona intestinalis	N	N	99
	Clavelina lepadiformis	N	Y	98
	Corella eumyota	Y	N	91
	Didemnid sp.	N	Y	50
	Diplosoma listerianum	N	Y	100
	Molgula sp.	N	N	73
	Morchellium argus	N	Y	6
Bryozoans	*Bowerbankia* sp.	N	Y	35
	Bugula fulva	N	Y	32
	Bugula neritina	Y	Y	69
	Callopora dumerilii	Y	Y	19
	Celloporella hyalina	N	Y	6
	Conopeum sp.	N	Y	24
	Cryptosula pallasina	N	Y	46
	Electra pilosa	N	Y	72
	Cheilostome bryozoan species A	N	Y	1
	Membranipora membranipora	N	Y	2
	Tricellaria inopinata	Y	Y	97
	Watersipora subtorquata	Y	Y	31
Crustaceans	*Balanus crenatus*	N	N	75
	Balanus perforatus	N	N	23
	Austrominius modestus	Y	N	20
	Verucca stroemia	N	N	1
Cnidarians	*Anemone* sp.	N	N	13
Molluscs	*Anomia* sp.	N	N	41
	Mytilus sp.	N	N	12
Polychaetes	*Spirobranchus* sp.	N	N	100
	Spirorbis spirorbis	N	N	13
Sponges	Sponge sp.	N	Y	7
