# Supplementary material for: Disentangling the impacts of heat wave magnitude, duration and timing on the structure and diversity of sessile marine assemblages
Source: PeerJ. 2015 Mar 26;3:e863. doi: 10.7717/peerj.863 (PMC4380158; doi:10.7717/peerj.863)
Supplement: Table S2 — Univariate permutational ANOVAs to test for differences in assemblage level metrics between HW Timings and Magnitudes, based on final values of total abundance (a,b), species richness (c,d) and dry weight biomass (e,f). Permutations (4999 under a reduced model) were based on Euclidean distances between untransformed data. Main tests were conducted on both the 1-week (a,c,e) and 2-week (b,d,f) HW duration experiments separately. Significant P values (at P < 0.05) are shown in bold. Significant HW Magnitude effects were investigated further with post-hoc pairwise comparisons. [file peerj-03-863-s006.pdf]

Table S2. Univariate permutational ANOVAs to test for differences in assemblage level metrics between HW Timings and Magnitudes, based on final values of total abundance (a,b), species richness (c,d) and dry weight biomass (e,f). Permutations (4999 under a reduced model) were based on Euclidean distances between untransformed data. Main tests were conducted on both the 1-week (a,c,e) and 2-week (b,d,f) HW duration experiments separately. Significant P values (at P < 0.05) are shown in bold. Significant HW Magnitude effects were investigated further with post-hoc pairwise comparisons.

(a) Total abundance – 1 week HW duration experiment

Source	df	SS	F	P
HW Magnitude	3	49424	2.59	0.063
HW Timing	2	768280	60.44	**0.001**
Ti x Ma	6	15791	0.41	0.85
Residual	52	330480		
Total	63	1149200		

(b) Total abundance – 2 week HW duration experiment

Source	df	SS	F	P
HW Magnitude	3	466090	18.26	**0.001**
HW Timing	2	125410	73.73	**0.001**
Ti x Ma	6	105810	2.07	0.098
Residual	52	442220		
Total	63	2209700		

Post-hoc tests for HW Magnitude : FC ≠ C = T1 = T2

(c) Species richness – 1 week HW duration experiment

Source	df	SS	F	P
HW Magnitude	3	43.01	2.89	0.055
HW Timing	2	207.09	20.94	**0.001**
Ti x Ma	6	20.91	0.70	0.645
Residual	52	257.12		
Total	63	523.75		

(d) Species richness – 2 week HW duration experiment

Source	df	SS	F	P
HW Magnitude	3	121.45	10.34	**0.001**
HW Timing	2	264.36	33.77	**0.001**
Ti x Ma	6	19.90	0.85	0.542
Residual	52	203.52		
Total	63	602.44		

Post-hoc tests for HW Magnitude : FC ≠ C = T1 = T2

(e) Total biomass – 1 week HW duration experiment

Source	df	SS	F	P
HW Magnitude	3	23.81	1.55	0.196
HW Timing	2	531.18	51.88	**0.001**
Ti x Ma	6	48.16	1.57	0.172
Residual	52	266.16		
Total	63	849.81		

(f) Total biomass – 2 week HW duration experiment

Source	df	SS	F	P
HW Magnitude	3	10.19	0.44	0.735
HW Timing	2	497.91	32.59	**0.001**
Ti x Ma	6	63.42	1.38	0.225
Residual	52	397.19		
Total	63	953.28		
