# Supplementary material for: Disentangling the impacts of heat wave magnitude, duration and timing on the structure and diversity of sessile marine assemblages
Source: PeerJ. 2015 Mar 26;3:e863. doi: 10.7717/peerj.863 (PMC4380158; doi:10.7717/peerj.863)
Supplement: Table S3 — One-way univariate permutational ANOVAs to test for the effects of HW Magnitude on the abundance of dominant native species. Abundance values reflected the relative change in actual abundance between the colonisation period (Phase 1) and the end of the experiment (Phase 3), and are shown graphically in Fig. 5. Permutations (999 unrestricted) were based on Euclidean distances between untransformed change in abundance (plus 1,000). Significant P values (at P < 0.05) are shown in bold. [file peerj-03-863-s007.pdf]

Table S3. One-way univariate permutational ANOVAs to test for the effects of HW Magnitude on the abundance of dominant native species. Abundance values reflected the relative change in actual abundance between the colonisation period (Phase 1) and the end of the experiment (Phase 3), and are shown graphically in Fig. 5. Permutations (999 unrestricted) were based on Euclidean distances between untransformed change in abundance (plus 1000). Significant P values (at $P < 0.05$) are shown in bold.

Species	HW duration	Timing	Magnitude F	Magnitude P	Pairwise
Ciona intestinalis	1 week	1	0.53	0.594	-
		2	2.59	0.115	-
		3	1.07	0.383	-
	2 week	1	1.13	0.389	-
		2	0.80	0.551	-
		3	1.28	0.183	-
Botryllus schlosseri	1 week	1	6.95	**0.008**	C = T1 > T2
		2	7.06	**0.006**	C > T1 = T2
		3	2.89	0.099	-
	2 week	1	1.13	0.374	-
		2	0.17	0.855	-
		3	1.28	0.147	-
Electra pilosa	1 week	1	0.13	0.886	-
		2	0.35	0.753	-
		3	1.07	0.398	-
	2 week	1	3.40	**0.044**	C = T1 > T2
		2	0.59	0.614	-
		3	0.08	0.940	-
Balanus crenatus	1 week	1	2.83	0.090	-
		2	2.36	0.110	-
		3	0.38	0.715	-
	2 week	1	2.25	0.118	-
		2	0.43	0.775	-
		3	1.28	0.159	-
